# Development and Characterisation of a Microneedle Sensor for Intrapartum Fetal Monitoring

**DOI:** 10.3390/bios15080517

**Published:** 2025-08-08

**Authors:** J. M. Mitchell, C. V. Thatte, R. Sebastian, C. O’Mahony, R. A. Greene, J. R. Higgins, P. Galvin, F. P. McCarthy, S. R. Teixeira

**Affiliations:** 1 Department of Obstetrics and Gynaecology, University College Cork, T12YE02 Cork, Ireland; 2 Tyndall National Institute, T12R5CP Cork, Irelandryan.sebastian@tyndall.ie (R.S.); conor.omahony@tyndall.ie (C.O.); paul.galvin@tyndall.ie (P.G.); 3 Insight Research Ireland Centre for Data Analytics, Tyndall National Institute, University College Cork, T12R5CP Cork, Ireland; 4 INFANT Research Centre, T12YE0 Cork, Ireland

**Keywords:** intrapartum monitoring, pH, sensor, microneedle, labour, interstitial fluid, electrochemistry

## Abstract

This study presents the in vitro and preliminary ex vivo development of a novel microneedle-based pH sensor for continuous intrapartum fetal monitoring. The objective was to evaluate the feasibility of using microneedle sensors to monitor fetal pH during labour and to develop a proof-of-principle microneedle pH sensor that meets clinical requirements such as high sensitivity to small pH changes (0.05 units) within a relevant range (6.50–7.45), minimal tissue disruption, and a compact design suitable for transcervical placement on the fetal scalp (<40 mm diameter). Platinum microneedles were passivated with ArCare medical adhesive and coated with iridium oxide via electrodeposition. Sensitivity was tested in phosphate buffered saline (PBS) and artificial interstitial fluid (ISF), using both external Ag/AgCl and internal platinum pseudo-reference electrodes. In PBS, the sensor exhibited linear responses in increments of 0.05 pH units over the clinically relevant range (6.5–7.45), with slopes of −60.49 mV/pH (R^2^ = 0.946, accuracy = 97.65%) and −63.2 mV/pH (R^2^ = 0.910, accuracy = 93.70%) in the external and internal configurations, respectively. In ISF, a slope of −25.5 mV/pH (R^2^ = 0.979) was obtained. Ex vivo testing on human skin confirmed successful microneedle penetration without visible iridium oxide transfer or tissue damage, as indicated by methylene blue staining. These findings support the potential for continuous minimally invasive fetal pH monitoring during labour, representing a significant step toward more objective and specific intrapartum assessment.

## 1. Introduction

The aim of intrapartum fetal monitoring is to improve perinatal outcomes by identifying foetuses at risk of hypoxia while avoiding unnecessary obstetric intervention [1]. Cardiotocography (CTG), a method of fetal heart rate assessment, is employed in the majority of births globally but has not reliably demonstrated the ability to detect or prevent hypoxic brain injury in the foetus [2,3,4,5,6,7]. Although CTG use may reduce the incidence of neonatal seizures, it has also been associated with a higher rate of Caesarean deliveries compared with intermittent auscultation, without meaningful improvements in other neonatal outcomes [8]. Its clinical utility is further limited by variability in interpretation between and within observers and by its poor positive predictive value for fetal hypoxia, which contributes to a high false-positive rate [2,9,10]. As a result, obstetric decisions may be influenced by overestimated risk, leading to interventions that may not be necessary. This dynamic likely plays a role in the global rise in Caesarean section rates, which increased from 20.7% to 32.1% in the United States between 1996 and 2021, and from 14.7% to 35.21% in the United Kingdom between the 1990s and 2021–2022 [11,12,13].

The use of fetal blood sampling as an adjunctive method of intrapartum monitoring does not reduce the number of Caesarean sections or the rate of neonatal encephalopathy compared with fetal heart rate monitoring alone [14]. It is a complex, invasive, and lengthy procedure that cannot demonstrate continuous trends in pH [2,15].

There is a need for better intrapartum fetal surveillance methods that will decrease the incidence of adverse neonatal and long-term neurodevelopmental outcomes while minimising unnecessary obstetric intervention. 

Microneedle sensors may offer a novel solution for non-invasive continuous monitoring of interstitial fluid (ISF) pH, which reflects blood pH and typically ranges from 7.35 to 7.45 under normal conditions [16,17]. Microneedle sensors could provide instantaneous, continuous, and objective data, allowing for the early recognition of fetal acidosis and immediate interventions if necessary [18,19]. They are associated with less pain and a lower risk of infection compared with hypodermic needles [20,21,22].

Microneedles have been developed as pH sensors for use in clinical settings, such as assessing for ischaemic tissue damage [23], and the measurement of pH in cerebrospinal fluid, urine, brain tissue, and cardiac tissue [24,25,26]. Garcia-Guzman et al. [18] characterised a potentiometric pH microneedle sensor for transdermal pH measurements in the interstitial space fluid. In their study, commercially available stainless steel solid microneedles were externally modified with an ion-selective membrane and tested in solutions ranging from pH 5.0 to 8.5, with increments of 0.5 pH units. The clinical decision-making thresholds for fetal scalp pH are normal (>7.25), subnormal (7.20–7.25), and pathological pH (<7.20) to guide intrapartum clinical decisions [27]. Given that diagnostic thresholds for fetal scalp pH rely on detecting differences of 0.05 pH for clinical decision making, it is important to demonstrate that a sensor being used for intrapartum management can accurately detect such small variations in pH [28]. 

This study aims to (i) evaluate microneedle pH sensors as a minimally invasive method for continuous fetal pH monitoring during labour and (ii) to characterise a proof-of-principle microneedle sensor with ArCare medical grade adhesive tape passivation and iridium oxide (IrOx) functionalisation under both in vitro and initial ex vivo conditions, capable of measuring pH levels ranging from 6.5 to 7.45, with increments of 0.05 pH, in interstitial fluid. Ultimately, this sensor is intended to be applied transcervically to the fetal scalp using a small patch. This work represents the initial step toward a minimally invasive technique for continuously monitoring fetal cranial dermal interstitial pH during labour. 

## 2. Materials and Methods

### 2.1. Reagents

Analytical grade reagents were used throughout this study. Oxalic acid (C_2_H_2_O_4_), calcium chloride (CaCl_2_), 4-(2-hydroxyethyl)-1-piperazineethanesulfonic acid (Hepes), potassium chloride (KCl), magnesium sulfate (MgSO_4_), sodium chloride (NaCl), monosodium phosphate (NaH_2_PO_4_), saccharose, magnesium chloride (MgCl_2_), sodium carbonate (Na_2_CO_3_), and 30% hydrogen peroxide (H_2_O_2_) were obtained from Sigma Aldrich. Iridium chloride hydrate (IrCl_4_-H_2_O) was purchased from Thermo Fisher Scientific (Waltham, MA, USA; formerly Alfa Aesar), and buffer pH solutions were procured from Merck (Darmstadt, Germany). Methylene blue and silver/silver chloride paste were purchased from Brunel Microscopes Ltd. and BAS Inc., respectively.

A 1 mM solution of ferrocene carboxylic acid (FCA) was prepared in 10 mM phosphate buffered saline (PBS), both purchased from Sigma Aldrich (Burlington, MA, USA). Artificial ISF was prepared by combining 5 mM CaCl_2_, 10 mM Hepes, 3.5 mM KCl, 0.7 mM MgSO_4_, 123 mM NaCl, 1.5 mM NaH_2_PO_4_, and 7.4 mM saccharose. This solution was adjusted to pH 7.5 in accordance with established protocols [19].

The IrOx was prepared following the method described by Yamanaka et al., 1989 [29]. IrCL_4_-H_2_O was dissolved in deionised water under magnetic stirring for 30 min. Then, 1ml of 30% H_2_O_2_ was added, and the solution was stirred for an additional 30 min. Subsequently, 0.5g of C_2_H_2_O_4_, was incorporated with continuous stirring for another 30 min, after which the pH was adjusted to 10.5 using Na_2_CO_3_. 

Microneedle wafers were fabricated using the Epotek 353ND kit (Parts A and B, Epoxy Technology). All solutions were prepared using double-distilled deionised water.

Methylene blue and silver/silver chloride paste were purchased from Brunel Microscopes Ltd. (Chippenham, UK) and Radionics (Dublin, Ireland), respectively. 

Ex vivo human skin samples, obtained following abdominoplasty procedures under full ethical approval from the Clinical Research Ethics Committee of the Cork Teaching Hospitals (ECM 4 (aa) 06/08/13), were stored at −80 °C until use. For experiments, the skin samples were sectioned into 35 × 35 mm^2^ pieces, with adipose tissue removed. Before mounting in a skin holder (to simulate natural skin tension and facilitate staining), the samples were rehydrated in 0.01 M PBS for fifteen minutes.

### 2.2. Equipment

Electrochemical measurements were carried out at room temperature using a Metrohm Autolab PGSTAT100 potentiostat with Nova software 2.1 (Metrohm, Cheshire, UK). The surface morphology of the iridium oxide coating was examined using a scanning electron microscope (FEI Quanta 650 FEG SEM; Hillsboro, OR, USA) with a working voltage of 5 kV. All experiments were conducted at room temperature within a Faraday cage, and pH values were verified using a commercial pH meter (FiveEasy Plus FP20, Mettler Toledo; Greifensee, Switzerland).

### 2.3. Electrochemical Characterisation of the Microneedles

**Figure 1:**
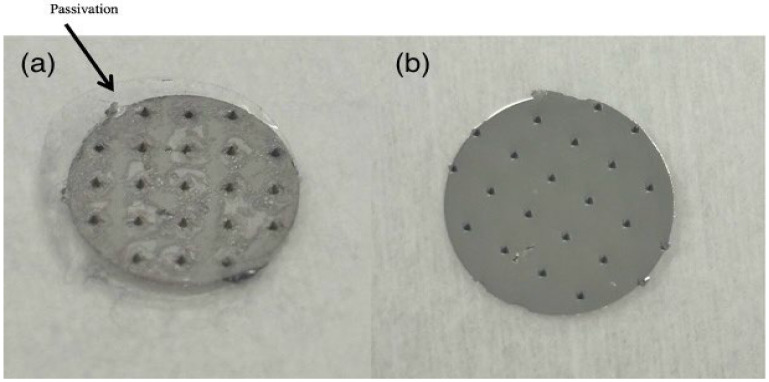
(**a**) Passivated platinum microneedle array; (**b**) unpassivated platinum microneedle array.

Solid microneedle sensor arrays were developed through micromoulding techniques, followed by a metallisation process to establish reference and working electrodes as previously described (Figure 1) [30,31]. Platinum was used as the electrode material due to its biocompatibility, chemical inertness, conductivity, and durability [32,33]. The sensors were passivated with ArCare 7759, a 55 μm thick medical-grade adhesive film from Adhesives Research (Limerick, Ireland) to prevent interference from the metallised areas on the microneedle sensor signal (Figure 1). The film was cut using a Graphtec CE7000-40 vinyl cutter into 9.5 mm diameter circles, each featuring 500 μm holes arranged to match the microneedle array pitch. The tape was aligned with the microneedles and manually pressed into place with forceps to ensure proper attachment to the substrate [34]. The needles were 500 μm tall with a tip-to-tip pitch of 1.75 mm.

### 2.4. Microneedle Electrode Cleaning

Microneedle arrays were cleaned using cyclic voltammetry (CV) in a solution of 10 mM PBS and 1 mM FCA. CV was performed over a potential range of −0.5 V to + 0.5 V at a scan rate of 0.05 V/s for 4 scans. A three-electrode setup was used, consisting of an MN array electrode as the working electrode, platinum wire as the counter electrode, and an Ag/AgCl reference electrode. An acid cleaning step was then carried out using H_2_O_2_, applying 10 scans from 0 to + 0.6 V at a scan rate of 0.05 V/s. This was followed by an additional 4 CV scans in the FCA solution to complete the cleaning process.

### 2.5. Electrodeposition of Iridium Oxide

The microneedle surface was modified with IrOx using an Autolab potentiostat (detailed above). IrOx was selected as the sensor material since it exhibits high sensitivity across a broad pH range, rapid response times, good chemical selectivity, and suitability for miniaturisation [35,36]. An anodic electrodeposition of an IrOx layer was achieved via standard electrochemical CV using 50 cycles. A three-electrode setup was used to deposit IrOx onto the working electrode, as described above. 

The potential was swept between −0.8 V and + 0.8 V at a scan rate of 0.1 mV/s. 

CV scans were used to calculate the electrochemical surface area of the sensors before and after IrOx deposition. To evaluate the stability of the sensors after IrOx deposition, CV scans in FCA were performed. The potential was varied from 0 to + 0.6 V at a scan rate of 0.05 mV/s, with measurements taken on days 1, 2, 5, 6, 8, 10, 12, 18, 28, 35, and 61 post-deposition.

### 2.6. Sensitivity and Stability of the pH Microneedle Sensors

**Figure 2:**
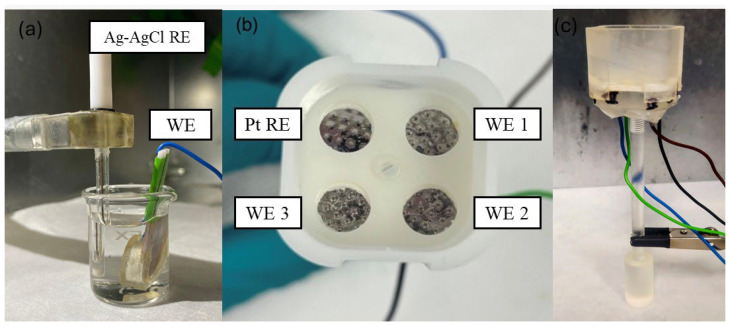
(**a**) External setup used; (**b**) internal setup with three passivated platinum working electrodes (WE) and one unpassivated platinum pseudo-reference electrode (RE) as viewed from above; (**c**) internal setup.

The sensitivity and stability of the sensors was evaluated by assessing their response to changes in PBS and artificial ISF with different pH levels ranging from 5.5 to 7.45. We altered the pH of the solutions by adding hydrochloric acid (HCl) or sodium hydroxide (NaOH) as demonstrated in the existing literature [37,38]. The pH values were validated with a commercial pH meter. Open-circuit potential (OCP) measurements were carried out at room temperature to evaluate the potential electrode pH response. We initially tested the stability and sensitivity of the sensors using an external setup with a two-electrode electrochemical cell, where a microneedle electrode served as the working electrode and an Ag/AgCl wire as the reference electrode (Figure 2a). The same set of sensors was used throughout the stability study, with measurements conducted on days 1, 5, 9, 15, 35, and 60 to assess their long-term performance in both PBS and ISF. Sensor sensitivity was then evaluated in PBS using an internal setup that employed an unpassivated platinum microneedle array as a pseudo-reference electrode and a passivated microneedle array as the working electrode (Figure 2b,c) [39,40,41]. The electrodes were mounted on a plastic holder, with each microneedle array spaced 1.9 mm apart. For measurements in artificial ISF, we began with an external setup. Next, we evaluated sensor sensitivity using an internal configuration with an unpassivated platinum pseudo-reference electrode. Finally, we repeated the sensitivity assessment using the same electrode treated with Ag/AgCl paste, similar to the existing published literature [18].

### 2.7. Interference Testing

Interferents were prepared by adding physiologically relevant concentrations—2 mM of MgCl_2_, 3.5 mM KCl, and 5 mM NaCl—to artificial ISF (pH 7.0), using the same concentrations as used in the existing literature [42]. OCP measurements were then performed in each solution, and the potential measured in ISF and the individual interferent solutions were compared. Dunnett’s test was conducted using SPSS version 28 to determine whether the mean potentials for the different solutions differed significantly from the control solution (pH 7.0).

### 2.8. Negative Control Experiment

A negative control experiment was performed to confirm that the IrOx modification was essential for pH sensing. We repeated the OCP calibration protocol in PBS using the same internal setup previously described. However, for this control, the working electrode was a bare passivated platinum microneedle that had not been modified with IrOx. The same pH range was tested, and the OCP readings were recorded under identical conditions to those used for the IrOx-modified electrodes.

### 2.9. Skin Testing

We immersed a skin sample in a PBS solution adjusted to pH 7.0 with HCl for 15 min. We then applied the microneedles to the skin using a high velocity microneedle applicator system (internal setup) and conducted three OCP measurements to determine the pH. A *t*-test was conducted to assess whether the mean potentials recorded from skin samples significantly differed from those obtained in vitro PBS experiments. After the OCP measurements, we examined the skin to assess for any transfer of IrOx from the microneedles. Finally, we applied a 1% w/v aqueous methylene blue solution to the treated area to assess the extent of microneedle penetration. The penetration efficiency was calculated by expressing the number of needles that penetrated as a fraction of the number of microneedles in the device.

## 3. Results and Discussion

### 3.1. Characterisation of the Electrodes

**Figure 3:**
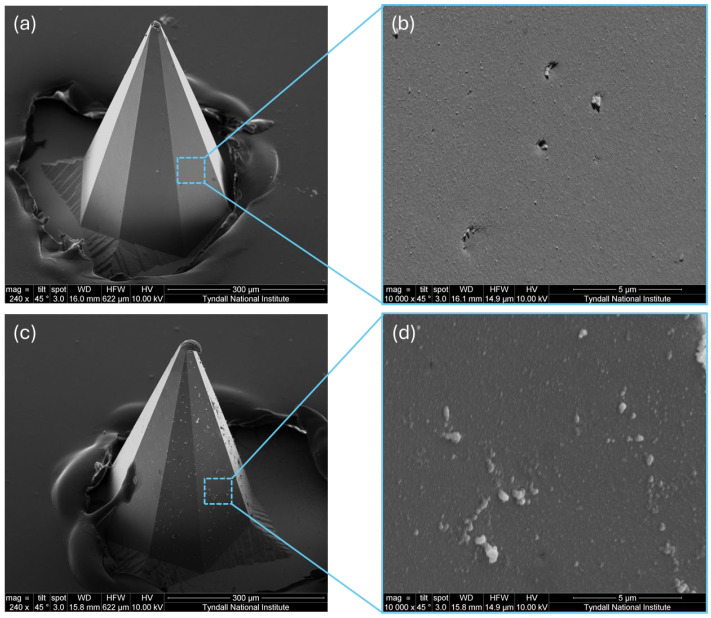
SEM images of microneedle tips before and after IrOx modification. (**a**) Unmodified platinum microneedle showing a smooth and clean surface; (**b**) higher magnification of the boxed region in (**a**), highlighting the low surface roughness of native Pt; (**c**) IrOx-modified microneedle exhibiting a visibly rougher surface due to the electrodeposited IrOx layer; (**d**) high magnification of the boxed region in (**c**), revealing a granular and porous morphology.

Microneedle pH sensors are characterised by sensitivity, accuracy, rapid response time, stability, and reproducibility. We evaluated these attributes and demonstrated the potential for the sensors to serve as an adjunct method for intrapartum fetal monitoring. Figure 3 compares the morphology of platinum microneedles with and without IrOx modification, both passivated using ArCare. Panels (a) and (b) show SEM images of the unmodified platinum microneedle. Panel (a) provides an overall view, while panel (b) offers a higher magnification of its relatively smooth surface. In contrast, panels (c) and (d) illustrate the IrOx-modified microneedle. Panel (c) presents the broader geometry, and panel (d) reveals the more textured surface arising from the IrOx layer. This modification is important because the IrOx layer enables pH sensing in interstitial fluid using solid microneedles [43]. It achieves this by facilitating reversible pH-sensitive redox reactions that accurately quantify hydrogen ion concentration [44]. The iridium oxide layer can be prepared using various methods, including electrodeposition, metal–organic chemical vapor deposition, sol–gel processing, and sputtering deposition [44,45]. Although sputtering deposition produces high-quality uniform IrOx films, it is expensive and offers limited control over the catalyst’s structural and morphological features [44]. In contrast, the sol–gel process is more cost-effective but often yields lower sensitivity due to its low porosity and reduced number of hydrophilic sites [46]. Electrochemical deposition, however, presents a cost-effective alternative by enabling the fabrication of IrOx films through the electrolysis of iridium-complex-containing solutions. This method is particularly advantageous because it allows for precise control over the IrOx loading and surface oxidation states by fine-tuning variables such as solution composition, current density, and deposition time, without requiring access to a cleanroom [45,47,48,49].

The surface roughening introduced by the IrOx deposition increases the electroactive area and enhances the sensor’s pH responsiveness by facilitating proton exchange at the electrode interface.

### 3.2. Electrodeposition of the Iridium Oxide Layer

**Figure 4:**
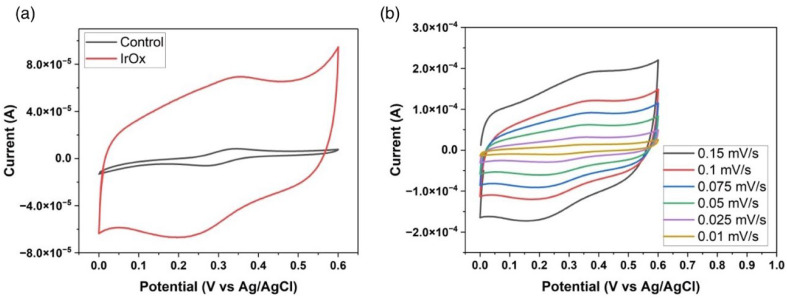
(**a**) Cyclic voltammetry analysis of the IrOx layer formation via electrodeposition; (**b**) cyclic voltammograms at varying scan rates for surface area evaluation.

Figure 4a shows the CV analysis during the electrodeposition of the IrOx layer. The passivated electrode initially exhibits an electrochemical surface area of 5.54 mm^2^, which increases by a multiplication factor of 12.23 to 67.74 mm^2^ after IrOx modification. This enhancement increases the number of active catalytic sites, allowing faster electron transfer and better electrode kinetics. This is critical for boosting the performance of electrochemical devices [50]. Before IrOx, the oxidation peak occurs at 0.313 V with a current of 1.28 × 10^−5^ A, and the reduction peak is observed at 0.264 V with a current of −1.22 × 10^−5^ A, yielding a peak separation (ΔEp) of ~49 mV. After IrOx deposition, the oxidation peak shifts to 0.348 V with a current of 1.03 × 10^−4^ A and the reduction peak shifts to 0.215 V with a current of −9.41 × 10^−5^ A, corresponding to a ΔEp of ~133 mV. These changes quantitatively demonstrate how the increased surface area and modified electron-transfer kinetics enhance the sensor’s performance [50,51]. Figure 4b presents cyclic voltammograms recorded at various scan rates. Both the peak currents and charging currents increase with higher scan rates, indicating a larger double-layer capacitance. Collectively, these results confirm that the IrOx deposition not only expands the electrochemically active surface area but also enhances the electrode’s charge storage capacity, thereby improving the overall electron-transfer kinetics and performance of the sensor in terms of higher sensitivity, lower limit of detection, faster response times, and a wider linear dynamic range, because of the increased number of active catalytic sites, larger double-layer capacitance, and more rapid electron-transfer rates [51].

**Figure 5:**
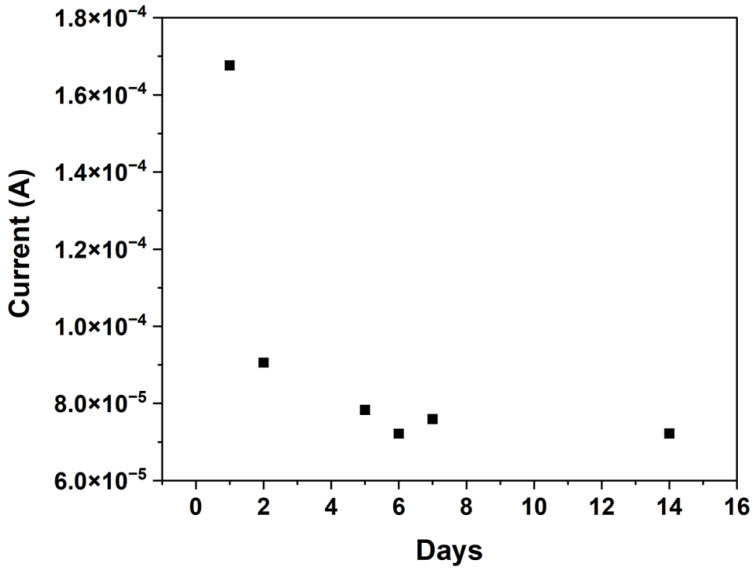
Stabilisation of iridium oxide over time (peak current vs. days).

Consistent with earlier reports [52,53], the IrOx film in our study required a stabilisation period following electrodeposition, reflecting hydration-driven structural rearrangements and ongoing oxidation processes. The peak current was observed to evolve until reaching a steady state, with our sensors exhibiting a stabilised peak current by day 5 post-deposition, as demonstrated in Figure 5. Therefore, sensor sensitivity testing began on day 5 after the IrOx layer was deposited. This conditioning is essential, ensuring the completion of structural reorganisation within the IrOx layer and resulting in reproducible and reliable sensor performance [52,53].

### 3.3. Sensitivity and Stability

**Figure 6:**
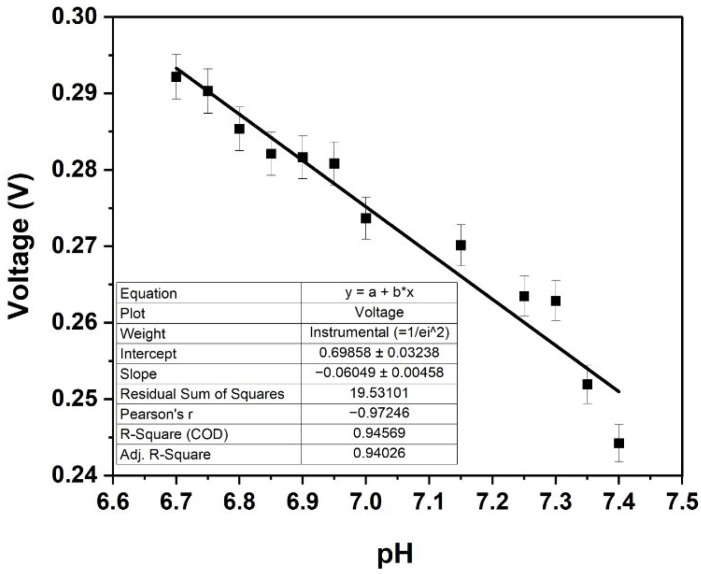
OCP-based calibration curve in external setup: voltage vs. pH in phosphate buffered saline (n = 6).

The sensors exhibited a linear response. Using the external setup in PBS across a pH range of 6.7 to 7.4 (in increments of 0.05), the sensors provided a linear Nernstian response of −60.49 mV/pH with a high correlation coefficient (R^2^) of 0.972 and an accuracy of 97.65% (Figure 6).

**Figure 7:**
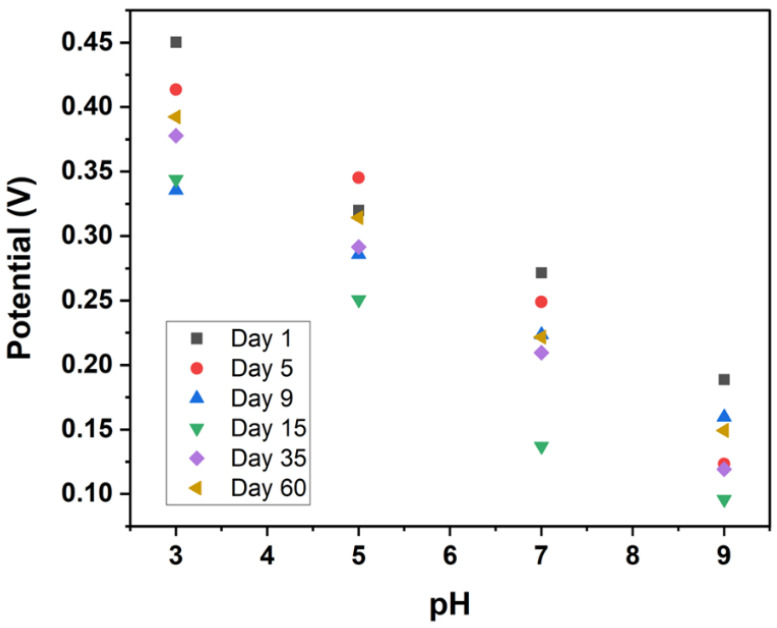
OCP stability of microneedle sensors in PBS from day 1 to day 60.

Figure 7 illustrates the OCP measurements of the microneedle sensors in PBS across a pH range of 3 to 9, measured on days 1, 5, 9, 15, 35, and 60. The data show that the potential response remains stable over time, with consistent changes in potential corresponding to alterations in pH. This indicates good long-term stability of the microneedle sensors. Furthermore, the ability to store these sensors in a dry state enhances their suitability for medical applications. Previous studies have reported that IrOx sensors require a one-hour buffer soak following dry storage, a procedure that is less than ideal in high-activity high-acuity clinical settings such as a labour ward [54].

**Figure 8:**
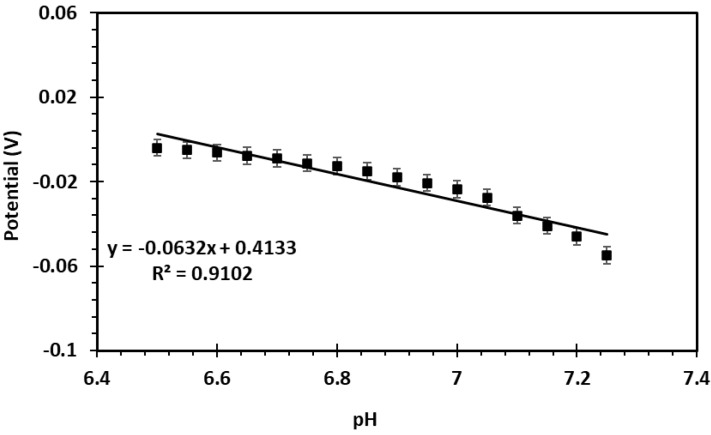
OCP-based calibration curve in internal setup: voltage vs. pH in phosphate buffered saline (n = 4).

When testing the internal setup in PBS over a pH range of 6.5 to 7.35 (in increments of 0.05), a sensitivity of −63.2 mV/pH (R^2^ = 0.91) and an accuracy of 93.07% were achieved (n = 4, Figure 8). Although the calibration curve yielded an R^2^ of 0.91, this was considered acceptable within the narrow physiological pH range assessed. Alternative regression models (e.g., second-order polynomial) were explored but did not provide meaningful improvements without overfitting, and a linear fit was retained for interpretability and consistency with previous pH sensor studies. 

Although the sensor demonstrated an in vitro accuracy of 93.07%, this level of precision is considered acceptable for a proof-of-concept study and is sufficient to detect clinically relevant pH changes as small as 0.05 units. Future development will aim to refine the device and achieve accuracy levels exceeding 95%, ensuring it meets the safety and reliability standards required for clinical use in fetal monitoring.

**Figure 9:**
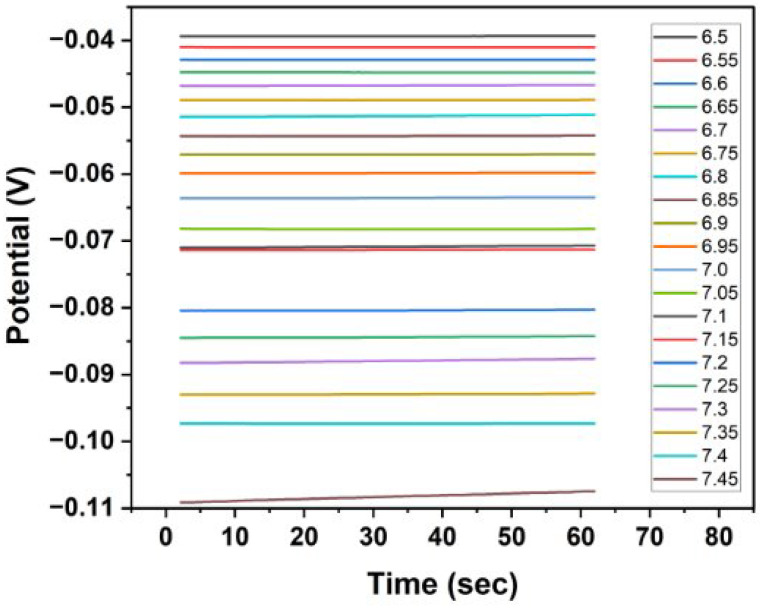
OCP vs. time for microneedle pH sensors in PBS at various pH values.

Figure 9 illustrates the real-time OCP readings of the sensor in PBS at multiple pH values ranging from 6.5 to 7.45. Each coloured trace represents the sensor response at a distinct pH, showing stable plateaus with minimal drift over the 60 s measurement period. The clear separation between the potential levels at each pH demonstrates the sensor’s sensitivity to small changes in pH. 

**Figure 10:**
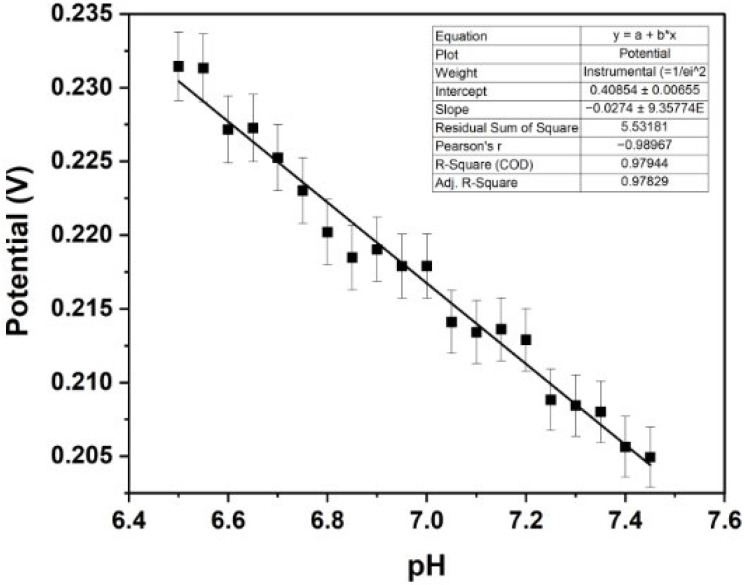
OCP-based calibration curve in internal setup: voltage vs. pH in artificial interstitial fluid using an Ag/AgCl paste applied to a platinum pseudo-reference electrode (n = 6).

Using an Ag/AgCl-modified platinum pseudo-reference electrode, the sensor achieved a highly linear calibration in artificial ISF, with a slope −27.5 mV/pH and R^2^ of 0.979, affirming the sensor’s capability to function in a complex medium that simulates the composition of natural ISF (Figure 10). Despite the reduced sensitivity in ISF, the sensor maintained the ability to detect pH changes as small as 0.05 units, which falls within the clinically relevant range for fetal monitoring applications.

The initial use of an unpassivated platinum pseudo-reference electrode in ISF likely led to a non-linear slope due to its inherent instability in complex media [55]. In complex solutions like ISF, various ions and impurities can interact with the unpassivated platinum surface, leading to the uncontrolled formation or dissolution of surface oxide layers. This can result in fluctuating potentials rather than a stable baseline [55]. Additionally, without a well-defined redox equilibrium, the electrode potential may drift during the measurement, further contributing to the non-linear behaviour observed [55]. We coated the platinum pseudo-reference electrode with an Ag/AgCl paste as performed in the similar existing research [18]. Ag/AgCl is the most frequently used reference electrode due to its high stability and non-toxicity [56]. Coating the electrode with Ag/AgCl paste helped establish a more stable well-defined potential through the formation of an insoluble salt layer, which minimised these variations and yielded a linear response [57]. Notably, the sensitivity of the sensor was lower in ISF than in PBS. This reduction in sensitivity is likely due to the more complex ionic composition of ISF [58]. Although the decreased sensitivity in ISF poses a limitation when translating our in vitro findings to a physiological context, the maintained linearity and high correlation coefficient in both media demonstrate the sensor’s potential. These findings provide a valuable foundation for future optimisation and in vivo validation. To address the potential long-term drift of unpassivated Pt pseudo-reference electrodes in ISF, future work will explore alternative coatings such as Ag/AgCl layers or polymer-based membranes to enhance electrochemical stability in complex biofluids.

### 3.4. Interference

**Figure 11:**
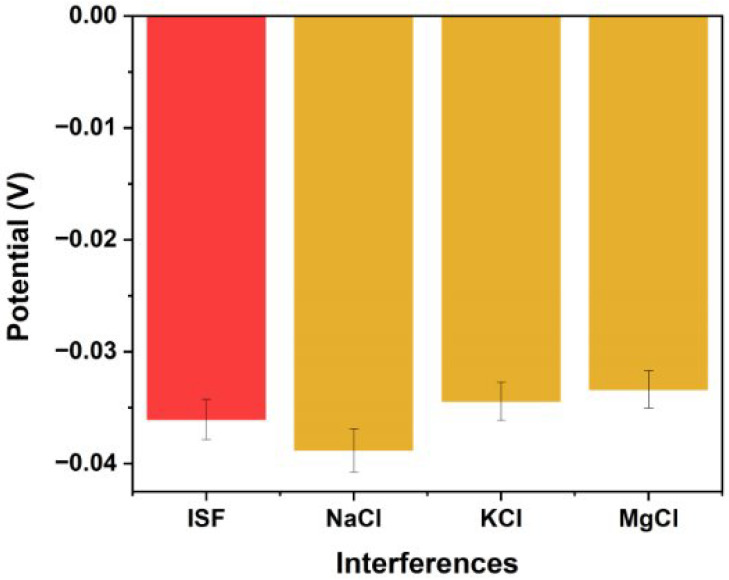
Potential in ISF vs. solutions with physiologically relevant interferents (pH 7.0).

At pH 7.0, the sensor measured an average potential of −0.03606 ± 0.0186 V in ISF. In the presence of 5 mM NaCl, 3.5 mM KCl, and 2 mM MgCl_2_, the average potentials (n = 6) were −0.03883 ± 0.0209 V, −0.03444 ± 0.0207 V, and −0.03339 ± 0.0194 V, respectively. The difference in the mean potential for each solution was not statistically significant to the potential measured in ISF (*p* = 0.543, *p* = 0.842, *p* = 0.889, respectively). Figure 11 illustrates these results, with the bar chart showing comparable potentials across all tested solutions. Table 1 details the selectivity coefficient differences between each solution, which did not differ significantly. The sensor’s response remains consistent across ISF solutions containing NaCl, KCl, or MgCl_2_, indicating that these ions do not contribute to the calibration curve discrepancies observed with the unpassivated platinum microneedle array RE. Maintaining high selectivity is critical when analysing dynamic fluids like ISF, where the concentrations of various ions and compounds fluctuate continuously. Our results, which demonstrate negligible interference from NaCl, KCl, and MgCl_2_, are consistent with previous studies reporting minimal impact from these salts [18,42]. To ensure the selective detection of ISF pH during intrapartum application, the microneedle sensor is designed to access sub-stratum corneum interstitial fluid while minimising exposure to external fluids through skin-conforming barriers; future development will incorporate selective membranes to further mitigate interference from blood or amniotic fluid. 

### 3.5. Negative Control Experiment

**Figure 12:**
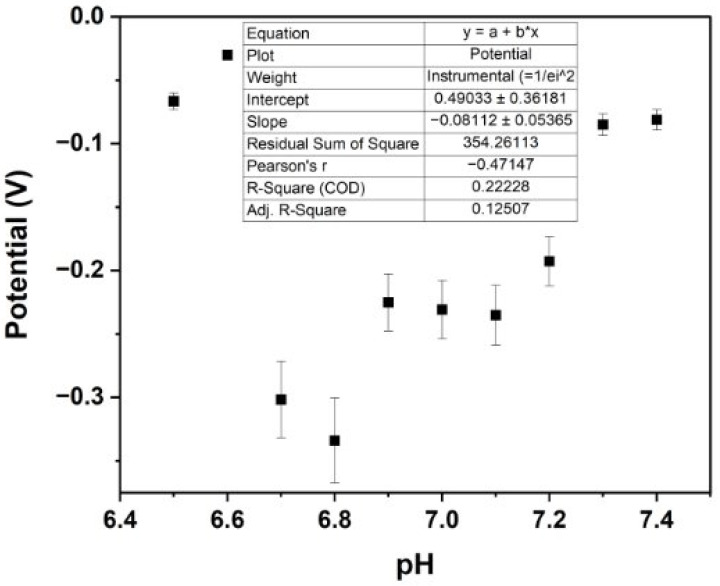
OCP calibration of unmodified platinum microneedle in PBS (negative control).

Unmodified platinum microneedles were used as a negative control to establish a baseline electrochemical response, enabling the specific contribution of the surface functionalisation to be accurately assessed. The OCP measurements obtained from the unmodified platinum microneedle did not yield a linear response across the tested pH range (Figure 12). This lack of linearity indicates that the sensor was not effectively measuring pH without the IrOx layer. Consequently, these negative control results demonstrate the importance of IrOx modification in achieving accurate and stable pH sensing.

### 3.6. Ex Vivo Skin Testing: Evaluating Microneedle Sensor Performance and Tissue Integrity

**Figure 13:**
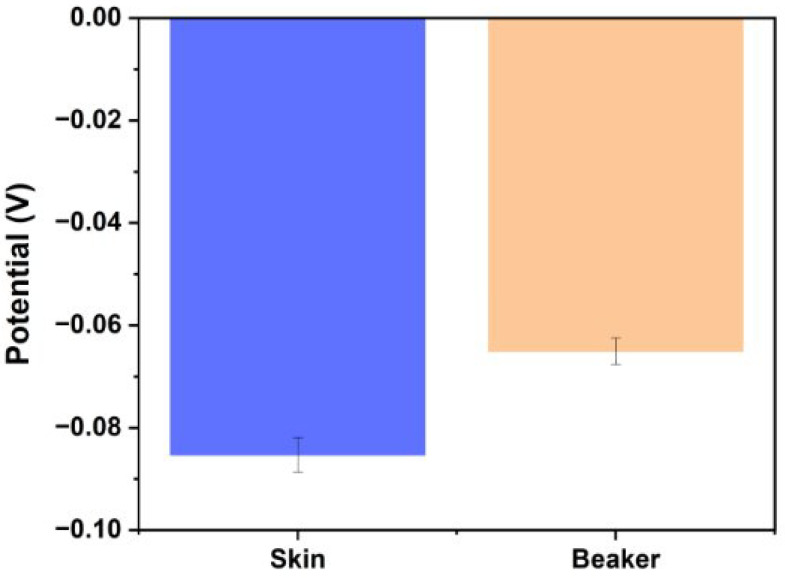
Comparison of OCP measurements in skin and PBS at pH 7.0.

Three microneedle sensors were tested on skin pre-soaked in PBS (pH 7.0), yielding an average potential of −0.0853 mV, whereas the mean potential measured for the same pH in PBS solution was −0.0651 (Figure 13). The difference between the mean potential measured in PBS solution and in skin preconditioned to pH 7.0 was not statistically significant (*p* = 0.342). Future studies could replicate these OCP measurements using the Ag/AgCl-coated platinum pseudo-reference electrode and consider extending the skin pre-soaking time in the pH 7.0 solution beyond 15 min. Notably, Chen et al. [59] soaked porcine skin in various pH solutions for 12 h when conducting similar experiments.

**Figure 14:**
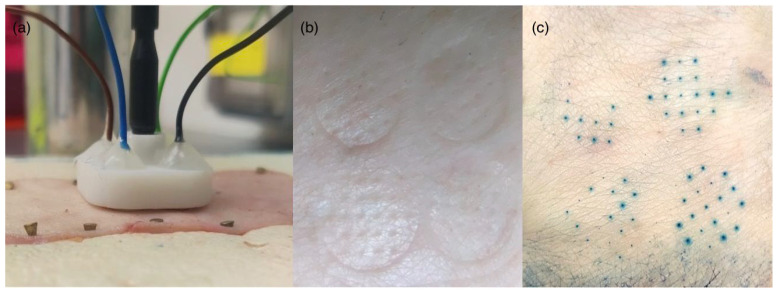
(**a**) Microneedle application; (**b**) skin examination; (**c**) methylene blue penetration test.

Visual inspection of the skin after microneedle application showed no visible transfer of IrOx, indicating that the sensor coating remained intact. Furthermore, methylene blue staining confirmed microneedle penetration without significant tissue disruption (Figure 14a), demonstrating the feasibility of using these microneedles for in-skin measurements (Figure 14b). Ex vivo testing was conducted on adult abdominal skin, which, although not identical to neonatal tissue, served as a consistent and ethically sourced model for preliminary evaluation. The use of a single donor batch reduced sample variability. Notably, the skin of term neonates shares similarities with adult skin, including a well-developed stratum corneum and comparable thickness, supporting the relevance of this model for assessing pH sensing in the dermal interstitial fluid of neonates [60,61,62]. This combination of retained coating integrity, minimal tissue disruption, age-independent skin compatibility, and compact dimensions demonstrates the sensor’s suitability for intrapartum monitoring. The penetration efficiency was calculated as 82%, in keeping with values reported in the existing literature [63].

This sensor is designed to be inserted vaginally and placed transcervically on the fetal scalp once the membranes have ruptured, either spontaneously or by amniotomy, during the active first stage or second stage of labour. The active first stage begins at 3–4 cm of cervical dilation (the end of the latent first phase) and continues until full dilation (10 cm), while the second stage spans from full dilation to delivery of the foetus [64]. Existing intrapartum fetal monitoring methods include fetal blood sampling, as discussed in the introduction. Amnioscopes used for fetal blood sampling range from 18 mm to 33 mm in external diameter [65,66]. Each of our microneedle arrays measures just 9.5 mm in diameter, and when combined, using an unpassivated platinum microneedle as the pseudo-reference electrode and a passivated platinum microneedle array as the working electrode, the total diameter is only 19 mm. These were spaced 1.9 mm apart on the holder. This compact size makes the device ideally suited for intravaginal placement during labour. Collaboration with mechanical engineers will be essential to refine this design into a clinically viable intrapartum monitoring tool that is easy to use within the restrictive anatomical environment and prevents amniotic fluid from interfering with the dermal interstitial fluid pH measurement. Microneedle arrays are classified as minimally invasive [67]. Their placement on the fetal scalp during labour follows procedures comparable to standard fetal monitoring techniques such as fetal heart rate monitoring via a fetal scalp electrode. As this placement does not involve any incisions and uses an anatomical orifice, the vagina, and dilated cervix, it can also be classified as minimally invasive [68]. Nonetheless, further evaluation of safety, tolerability, and potential risks will be undertaken in future clinical validation studies.

### 3.7. Comparison with the Existing Literature

The sensitivity of the microneedle sensor in PBS aligns with those found in the literature [18,26,69]. As mentioned previously, Lee et al. [23] developed a pH sensor to monitor tissue damage associated with ischaemia by electrochemically depositing PANI and Ag/AgCl onto an open window in the microneedle. These sensors achieved a sensitivity of 94 mV/pH in PBS. Zhou [26] created a PANI-functionalised acupuncture-based pH sensor for real-time cerebral monitoring (slope: −51.2 mV/pH in pH buffer solution, −46.8 mV/pH in serum). Mani et al. [24] developed a sensor using zinc oxide thin film on tungsten microneedles for cerebrospinal fluid and urine pH and reported a slope of −46.35 mV/pH in pH buffer solution. PANI has high electrical conductivity and biocompatibility; however, there are limitations in the use of PANI, such as its low processability and degradability [70,71]. In contrast with these existing works, we specifically focused on detecting small pH increments within the clinically relevant range for fetal hypoxia, and incorporated ArCare passivation and IrOx deposition. As previously noted, IrOx is an excellent choice for biosensor development because of its proven stability, conductivity, durability, and biocompatibility [54,72]. Zuliani et al. [25] designed an IrOx-functionalised sensor to map pH distributions in the rat heart; however, their approach used a gold-coated working electrode rather than a platinum one and did not demonstrate the capability to detect small increments in pH. Garcia-Guzman et al. characterised a potentiometric pH microneedle sensor for transdermal pH measurements in ISF, using commercially available stainless steel solid microneedles modified with an ion-selective membrane, with testing performed over a broader pH range (5.0 to 8.5) and in larger increments (0.5 pH units) [18]. Given that clinical decision making during intrapartum fetal monitoring relies on detecting differences as small as 0.05 pH units, our work demonstrates the importance of designing sensors capable of capturing such subtle changes to ensure accurate and timely obstetric intervention [28]. 

A significant strength of this work is the demonstration of a minimally invasive continuous monitoring system, validated through this in vitro and ex vivo study, that has the potential to improve the objectivity and specificity of intrapartum fetal monitoring. The multidisciplinary involvement of both obstetricians and chemical engineers facilitated the development of a sensor that exhibited appropriate sensitivity and accuracy within the clinical pH range, while meeting the user requirements of minimal tissue disruption, no transfer of IrOx from the sensor to the skin, compact size, and the capability for dry-state storage. 

The microneedle design could provide continuous measurement of intrapartum fetal pH, enabling trend analysis while potentially minimising both maternal and fetal discomfort, and reducing the risk of infection compared with fetal blood sampling [20,21,22]. The sensors exhibit high accuracy, sensitivity, and stability, with the capability to detect subtle pH variations that are essential for precise fetal monitoring during labour. Using a microneedle array as a pseudo-reference electrode simplifies the design of an intrapartum monitoring patch, as two small arrays can be combined into a single device that can be inserted vaginally, placed transcervically, and positioned on the fetal head. The successful integration of ArCare passivation to shield non-sensing areas, the effective electrodeposition of IrOx, and the use of an internal pseudo-reference electrode configuration establish a foundation for future in vivo testing.

Despite these promising results, several limitations still need to be addressed. First, inter-sensor variability was also observed, indicating that further refinement in the fabrication and passivation processes is necessary to ensure consistent performance. Second, the sensor was less sensitive in ISF using the internal setup; further modifications are required to improve sensitivity in complex media. Third, stability of the sensors was tested using an external setup with a two-electrode electrochemical cell, with a microneedle electrode serving as the working electrode and an Ag/AgCl wire as the reference electrode; this may have potentially improved the stability of the sensors. Finally, while this study demonstrates promising in vitro and initial ex vivo findings, further in vivo research is essential to fully evaluate sensor performance under physiological conditions. For example, an animal comparative crossover study, directly comparing the microneedle sensor readings with those of established blood gas analysers, could validate the sensor’s accuracy in a clinical setting and elucidate its potential to reliably monitor subtle pH variations in real time.

## 4. Conclusions

This work demonstrates a proof-of-principle microneedle-based pH sensor that achieves the sensitivity required for intrapartum fetal monitoring. The platinum microneedles, passivated with ArCare and functionalised with electrodeposited IrOx, exhibited a consistent linear response to small pH increments in both PBS and ISF using microneedle-based working and reference electrodes. The sensor showed a linear response (–63.2 mV/pH in PBS; −27.5 mV/pH in ISF) with an accuracy of 93.07% in PBS. It successfully penetrated ex vivo skin without any coating delamination, and minimal tissue disruption, confirming its suitability for continuous minimally invasive fetal pH measurement. The stability, compact size, and capability for dry-state storage were also demonstrated. These results highlight the sensor’s potential to provide precise and continuous fetal pH monitoring, which may reduce unnecessary obstetric interventions without increasing the incidence of neonatal encephalopathy. Further in vivo validation and device integration are necessary for clinical application. Beyond intrapartum pH monitoring, the microneedle-based platform demonstrated in this study offers a versatile foundation for adaptation to other analytes, including glucose, lactate, and electrolytes, supporting the future development of multiplexed minimally invasive biosensors for broader clinical applications.

## Figures and Tables

**Table 1:** Values of the selectivity coefficients between ions and the interference ions for pH sensor.

	Selectivity Value (V)	Selectivity Coefficient Difference (V)	*p*-Value
pH 7.0	−0.03606	Target ion	
NaCl	−0.03883	0.00277	0.543
KCl	−0.03444	0.00162	0.842
MgCl_2_	−0.03339	0.00267	0.889

## Data Availability

Data available on request.

## Abbreviations

The following abbreviations are used in this manuscript:

Ag/AgCl	Silver/silver chloride
CaCl_2_	Calcium chloride
CV	Cyclic voltammetry
CTG	Cardiotocograph
FCA	Ferrocene carboxylic acid
H_2_O_2_	Hydrogen peroxide
HCl	Hydrochloric acid
Hepes	4-(2-hydroxyethyl)-1-piperazineethanesulfonic acid
IrCl_4_·H_2_O	Iridium chloride hydrate
IrOx	Iridium oxide
ISF	Interstitial fluid
KCl	Potassium chloride
MgCl_2_	Magnesium chloride
MgSO_4_	Magnesium sulfate
Na_2_CO_3_	Sodium carbonate
NaCl	Sodium chloride
NaH_2_PO_4_	Monosodium phosphate
NaOH	Sodium hydroxide
OCP	Open-circuit potential
PBS	Phosphate buffered saline
